# Body mass index, waist circumference, and risk of hearing loss: a meta-analysis and systematic review of observational study

**DOI:** 10.1186/s12199-020-00862-9

**Published:** 2020-06-26

**Authors:** Jin-Rong Yang, Khemayanto Hidayat, Cai-Long Chen, Yun-Hong Li, Jia-Ying Xu, Li-Qiang Qin

**Affiliations:** 1 Department of Nutrition and Food Hygiene, School of Public Health, Soochow University, Suzhou, 215123 China; 2 Children’s Hospital of Soochow University, Suzhou, 215025 China; 3 State Key Laboratory of Radiation Medicine and Protection, School of Radiation Medicine and Protection, Soochow University, Suzhou, 215123 China

**Keywords:** Hearing loss, Obesity, Overweight, Body mass index, Waist circumference, Adiposity

## Abstract

**Background:**

Emerging evidence implicates excess weight as a potential risk factor for hearing loss. However, this association remained inconclusive. Therefore, we aimed to systematically and quantitatively review the published observational study on the association between body mass index (BMI) or waist circumference (WC) and hearing loss.

**Methods:**

The odds ratios (ORs) or relative risks (RRs) with their 95% confidence intervals (CIs) were pooled under a random-effects model. Fourteen observational studies were eligible for the inclusion in the final analysis.

**Results:**

In the meta-analysis of cross-sectional studies, the ORs for prevalent hearing loss were 1.10 (95% CI 0.88, 1.38) underweight, 1.14 (95% CI 0.99, 1.32) for overweight, OR 1.40 (95% CI 1.14, 1.72) for obesity, 1.14 (95% CI 1.04, 1.24) for each 5 kg/m^2^ increase in BMI, and 1.22 (95% CO 0.88. 1.68) for higher WC. In the meta-analysis of longitudinal studies, the RRs were 0.96 (95% CI 0.52, 1.79) for underweight, 1.15 (95% CI 1.04, 1.27) for overweight, 1.38 (95% CI 1.07, 1.79) for obesity, 1.15 (95% CI 1.01, 1.30) for each 5 kg/m^2^ increase in BMI, and 1.11 (95% CI 1.01, 1.22) for higher WC.

**Conclusions:**

In summary, our findings add weight to the evidence that elevated BMI and higher WC may be positively associated with the risk of hearing loss.

## Introduction

Hearing loss is a growing important global public health concern. In 2018, the World Health Organization (WHO) estimated there were 466 million (6.1% of the world’s population) people living with disabling hearing loss worldwide. The number of people with hearing loss is projected to increase to 630 million by 2030 and to 900 million in 2050 [1]. Between 1990 and 2016, hearing loss move up from the fifth to the third major cause of years lived with disability [2]. Hearing loss is not only restricting individuals’ ability to communicate and interact, but may also have a negative impact on physical and psychosocial well-being and quality of life of people with this disability [3–9]. Thus far, genetic predisposition, certain infections, ototoxic drugs, and longstanding exposure to excessive noise have been identified as risk factors for hearing loss [3, 10–12]. Considering that hearing loss may adversely affect physical, mental, and social well-being and quality of life, further identification of preventable or modifiable factors for hearing loss that could be useful to prevent or at least delay this condition should be a top public health priority.

Emerging evidence implicates excess weight [13] and its related cardiometabolic comorbidities, such as diabetes mellitus (DM) [14] and cardiovascular disease (CVD) [15–17], as potential risk factors for hearing loss. These associations are biologically plausible because individuals with these conditions are likely to have poor microvascular circulation that can lead to reduced blood supply to the cochlea, resulting in damage to the hair cells and eventually in hearing loss [12, 13, 18]. In the past few years, the findings of several epidemiological studies [19–28] have indicated that elevated body mass index (BMI), in the obesity range, and to a lesser extent, in the overweight range, was positively associated with hearing loss. However, the association between excess body weight and hearing loss remained inconclusive, as other studies found no association between both conditions [29–32]. Amid this uncertainty, some studies [19, 23] found that underweight was also positively associated with hearing loss.

Given these considerations, we aimed to perform a systematic review and meta-analysis of observational studies to provide further clarification of the potential association between BMI and hearing loss (primary aim). In addition, we also investigated the potential association between marker of central adiposity, waist circumference (WC), and hearing loss (secondary aim).

## Methods

### Search strategy

The present systematic review and meta-analysis followed the Meta-analysis Of Observational Studies in Epidemiology checklist [33]. Two investigators (J.-R.Y. and K.H.) independently performed the literature search, study selection, data extraction, and quality assessment.

The PubMed and Web of Science databases were used to identify the relevant articles in any language that were published from inception to May 2019. We used the following search terms to identify the relevant articles: (hearing OR hearing loss OR hearing impairment OR deaf OR deafness) AND (adiposity OR body fatness OR body size OR anthropometric OR body mass index OR waist circumference OR underweight OR overweight OR obesity). The references cited in the included studies were also scrutinized to identify additional relevant articles.

### Study selection

The observational studies of any design were selected for the final analysis only if they reported the risk estimates (relative risks (RRs), odds ratios (ORs), or hazard ratios (HRs)) with their corresponding 95% confidence intervals (CIs) of the association between BMI or WC and hearing loss in adults. Studies that specifically enrolled children or adolescents were excluded. If multiple articles reporting the data from the same population were identified, we included only those with the most up-to-date, complete, and relevant data.

### Data extraction and quality assessment

The following information were extracted from each included study: the first author’s last name, publication year, design, country, age of the participants, proportion of men, total sample and number of cases, exposure category, ascertainment of exposure, ascertainment of hearing loss, definition of hearing loss, and adjustment for confounders. The risk estimates from the maximally adjusted model were included whenever reported. The Newcastle-Ottawa Scale (NOS) [34] was used to judge the quality of the eligible studies. Three domains (selection, comparability, and outcome/exposure) were assessed by the NOS. The cut-off scores of 0–3, 4–6, and 7–9 correspond to low-, moderate-, and high-quality study, respectively.

### Statistical analysis

The ORs (from cross-sectional studies) or RRs (from longitudinal studies) with their 95% CIs were used as the measures of effect size. In the longitudinal study by Barrenas et al. [19], the data were expressed as ORs. However, given the rarity of hearing loss (4%) among the study population [19], the ORs were approximately equivalent to the RRs [35]. A DerSimonian and Laird random-effects model [36] was used to estimate the summary ORs or RRs with their 95% CIs for the association between BMI or WC and the risk of hearing loss. The subgroup and meta-regression analyses were not performed owing to the limited number of studies for each association of interest.

In the analysis of BMI, we first investigated the influence of abnormal BMI status (underweight as < 18.5 kg/m^2^, overweight as 25.0 to 29.9 kg/m^2^, and obesity as ≥ 30.0 kg/m^2^) as classified by the WHO [37] on the risk of hearing loss. If multiple BMI cut-offs that fell into one of the BMI categories as defined by the WHO, we then pooled the RRs or ORs under a fixed-effects model and used the pooled RRs or ORs for that BMI category. In addition, we further investigate the potential linear association between BMI and hearing loss by using the method proposed by Greenland and Longnecker [38] and Orsini et al. [39] to convert BMI categories into a continuous variable for each 5 kg/m^2^ increase in BMI.

In the analysis of WC, we only investigated the association between the highest versus lowest WC and hearing loss. The longitudinal study by Cruickshanks et al. [21] was not included in the meta-analysis of WC because the RR was expressed in a continuous variable for each 10 cm increase in WC.

Additionally, we performed sensitivity analysis by omitting a single study in each turn.

The *Q* and *I*^2^ statistics were used to determine the statistical heterogeneity across studies. For the *Q* statistic, *P* < 0.1 was considered as statistically significant; for the *I*^2^ statistic, the following cut-off points were used: < 25% (low heterogeneity), 25–50% (moderate heterogeneity), > 50–75% (high heterogeneity), and > 75% (severe heterogeneity) [40]. Publication bias was statistically assessed using Begg’s rank correlation and Egger’s regression test [41]. If publication bias was statistically significant, the trim and fill method was performed to adjust the bias [42]. All statistical analyses were performed using STATA software, version 11.0 (STATA Corp., College Station, TX, USA). All *P* values were two-sided, and the level of significance was set at < 0.05.

## Results

### Literature review and study characteristics

**Fig. 1:**
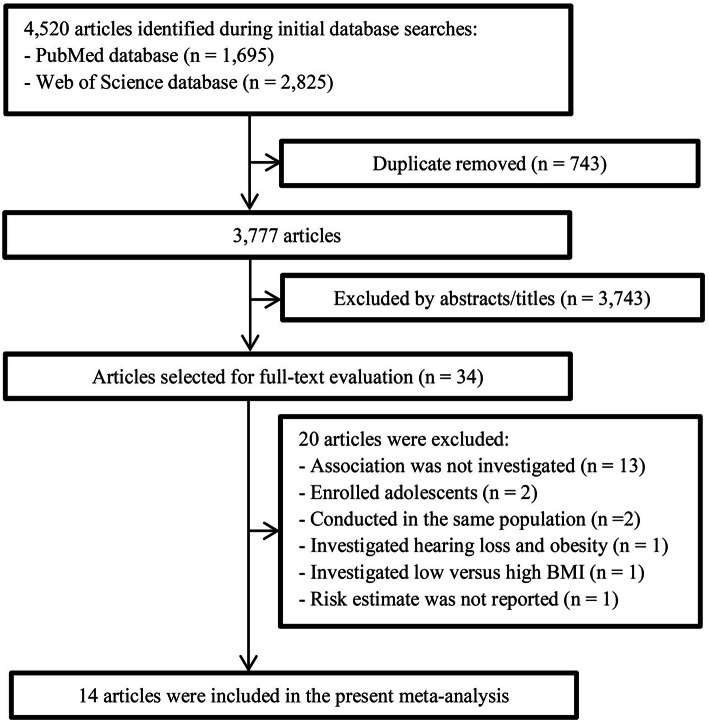
Flow-chart of the study selection process

The flow chart of study selection and the reasons for exclusion is presented in Fig. 1. During the initial database searches, a total of 4520 articles (1695 from PubMed and 2825 from Web of Science) were identified. After exclusion of duplicates and abstracts/titles screening, 34 articles were eligible for full-text evaluation. After full-text evaluation, 20 articles were further excluded. Of these excluded articles, 13 studies did not investigate the associations of interest, two studies [43, 44] enrolled adolescents, two studies [45, 46] were conducted in the same population as the included study [32], one study [47] comparing low versus high BMI (high BMI as reference), one study [48] investigating the association between hearing loss and obesity, and one study [49] did not report the risk estimate. Finally, 14 studies [19–32], with a total of 489,354 participants and 55,410 cases, were included in the final analysis.

### Study characteristics

Characteristics of the included studies are summarized in Additional file 1 (Table S1). Of the 14 included studies, six were longitudinal studies [19–21, 27–29] and eight were cross-sectional studies [22–26, 30–32]. Twelve studies [19–22, 24–29, 31, 32] were population-based and two studies [23, 30] were hospital-based. Occupation information was not adequately described in nearly all studies. The mean age of the participants was 44.8 years. By the sex of the participants, five [19, 22, 24, 29, 31] studies enrolled men only, two [20, 27] enrolled women only, and the remaining seven [21, 23, 25, 26, 28, 30, 32] included both men and women. Seven studies [19–22, 25, 27, 29] were conducted in Western countries (Australia, Finland, Sweden, and the USA), six [23, 24, 26, 28, 31, 32] in Asian countries (Bangladesh, China, Iran, Japan, and South Korea), and one [30] in African country (Nigeria). BMI or WC was self-reported in four studies [20, 22, 29, 31], while the remaining ten studies [19, 21, 23–28, 30, 32] relied on the data of measured BMI or WC. Hearing loss was measured by pure tone audiometry in 11 studies [19, 21–24, 26–28, 30–32] and self-reported in three studies [20, 25, 29]. The cut-points (frequencies and threshold) used to define hearing loss slightly varied across the studies. The WHO defines hearing loss as pure-tone thresholds of 25 dB or higher in the better ear or both ears [50]. Thus, the threshold of hearing loss among the included studies that used measured hearing loss was relatively comparable with that of hearing loss according to the WHO definition, with six studies [21, 24, 26, 27, 30, 32] used 25 dB for hearing loss threshold, three [19, 22, 31] used 20 dB as the threshold, one [23] used 26 dB as the threshold, and one [28] used 40 dB as the threshold. The information regarding the symmetry of hearing loss (unilateral or bilateral hearing loss) was not adequately described in nearly all studies. Thirteen of the 14 included studies were awarded at least seven stars, which indicated good quality studies (Additional files 2 and 3 Table S2-S3).

### Meta-analyses

#### Cross-sectional studies

**Fig. 2:**
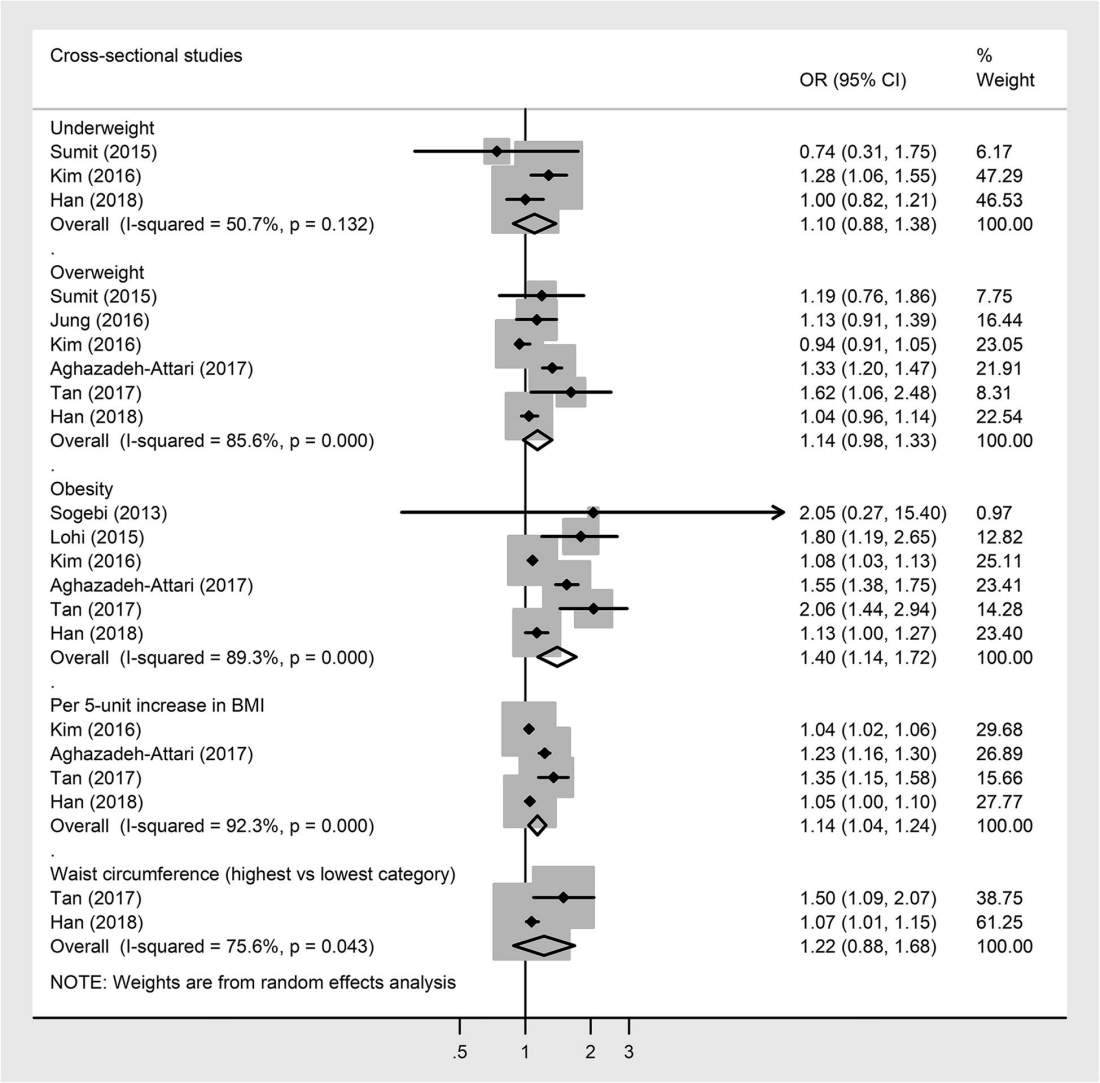
Cross-sectional studies investigating the association between BMI or WC and the odds of having hearing loss

Pooled cross-sectional studies revealed that obesity was positively associated with the odds of having hearing loss (OR 1.40, 95% CI 1.14, 1.72; Fig. 2) [22–26, 30]. However, underweight (OR 1.10 95% CI 0.88, 1.38; Fig. 2) [23, 26, 31], overweight (OR 1.14, 95% CI 0.99, 1.32; Fig. 2) [23–26, 31, 32], and higher WC (OR 1.22, 95% CO 0.88, 1.68; Fig. 2) [25, 26] were not associated with hearing loss. Each 5 kg/m^2^ increase in BMI [23–26] was associated with 14% increased odds of having hearing loss (Fig. 2). High heterogeneity was observed in all analyses (*I*^2^ ≥ 50.7). There was no evidence of publication bias in all analyses (all *P* Begg’s ≥ 0.17; all *P* Egger’s ≥ 0.18).

#### Longitudinal studies

**Fig. 3:**
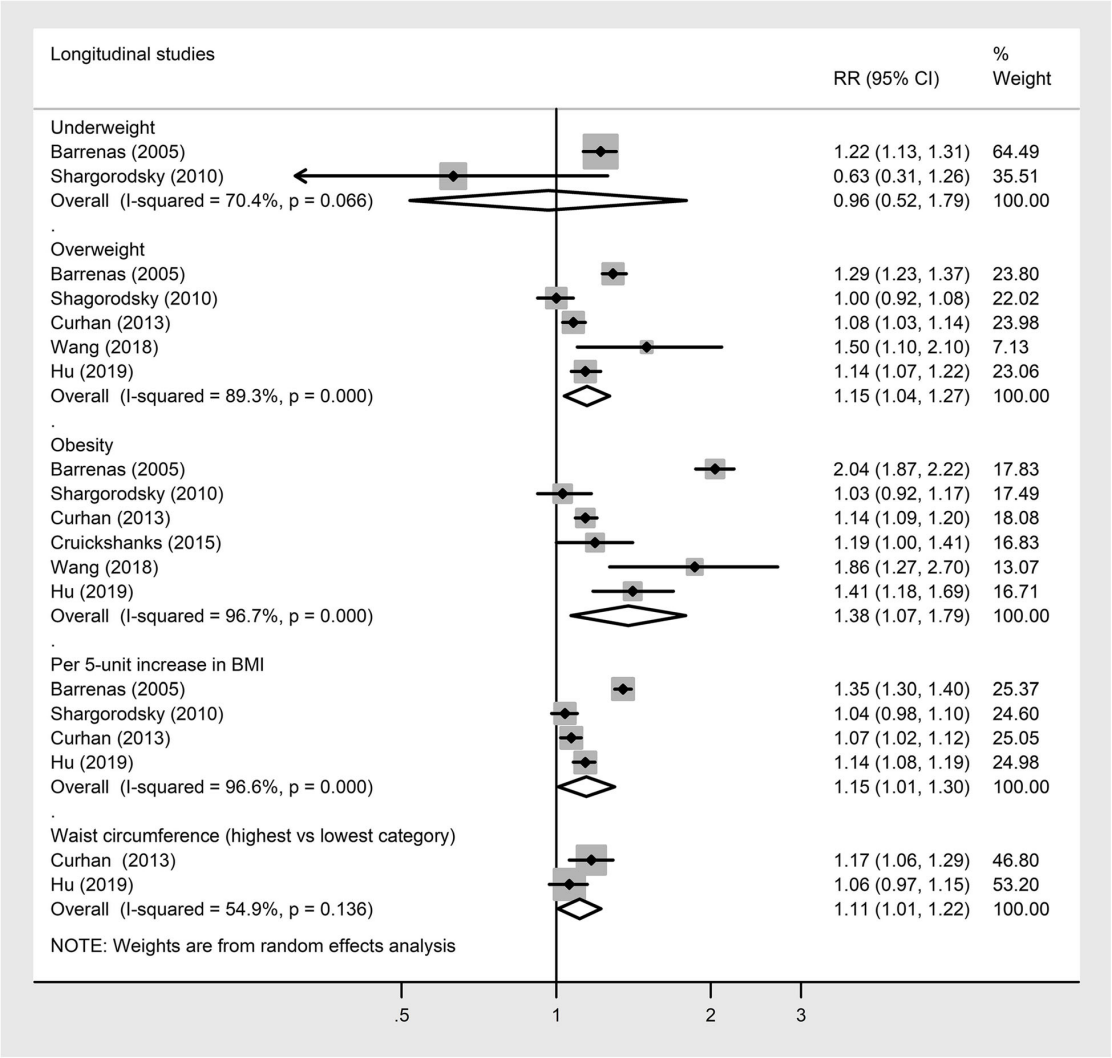
Longitudinal studies investigating the association between BMI or WC and the risk of hearing loss

Pooled longitudinal studies showed that overweight (1.15, 95% CI 1.04, 1.27; Fig. 3) [19, 20, 27–29], obesity (RR 1.38, 95% CI 1.07, 1.79; Fig. 3) [19–21, 27–29], and higher WC (RR 1.11, 95% CI 1.01, 1.22; Fig. 3) [20, 28] were associated with an increased risk of hearing loss. No association was observed between underweight [23, 26, 31] and the risk of hearing loss (RR 0.96, 95% CI 0.52, 1.79). Each 5 kg/m^2^ increase in BMI [19, 20, 28, 29] was associated with a 15% higher risk of hearing loss (Fig. 3). High heterogeneity was evident in all analyses (*I*^2^ ≥ 54.9). There was no evidence of publication bias in all analyses (all *P* Begg’s ≥0.19; all *P* Egger’s ≥ 0.12).

## Discussion

To the best of our knowledge, the present study is the first to systematically and quantitatively review the association between BMI or WC and hearing loss. Our findings indicated that elevated body mass index (BMI), particularly in the obesity range, and higher WC were positively associated with hearing loss. The findings of the positive association between elevated BMI and hearing loss were further supported by the linear relationship between BMI and hearing loss.

### Potential mechanisms

The underlying biological mechanisms by which excess weight may play a role in the development of hearing loss are poorly understood. Adipose tissue is an endocrine organ that secretes a number of pro-inflammatory and anti-inflammatory adipokines [51]. Excess adiposity promotes pro-inflammatory state that downregulates anti-inflammatory adipokines and upregulates pro-inflammatory adipokines [52], leading to vascular dysfunction and damage to the end-organs [53], cochlea in the case of hearing loss [54]. Adiponectin is an anti-inflammatory adipokine that may play important roles in the regulation of metabolism, insulin sensitivity, inflammation, and atherosclerosis [55]. Obesity is associated with low levels of plasma adiponectin [56]. Increased levels of plasma adiponectin have been shown to be inversely associated with a high-frequency hearing loss in human, suggesting that low levels of plasma adiponectin may potentially contribute to the development hearing loss [57]. The findings from two animal studies [58, 59] further support the potential role of adiponectin in the development of hearing loss. The first study [58] confirmed the expression of adiponectin receptor 1 in the auditory system of mice. Another study [59] showed that adiponectin deficiency exacerbated age-related hearing loss in knockout mice.

### Interpretation

Study design did not appear to alter the association between BMI and hearing loss, as the positive linear relationship between BMI and hearing loss were evident in both cross-sectional and cohort studies. However, two [20, 29] of the included longitudinal studies were relied on the data of self-reported BMI and hearing loss, although the authors of both studies indicated that self-reported BMI was validated and self-reported hearing loss could be a reliable assessment. Amid this assessment concern, the positive association between measured BMI and measured hearing loss was also evident in several longitudinal studies [19, 21, 27].

Age is a well-established risk factor for hearing loss, as hearing tends to gradually worsen with age [60]. Thus, it is possible that age may modify the positive association between overweight or obesity and hearing loss. Unfortunately, subgroup analyses according to age could not be performed owing to the limited number of studies. However, the majority of studies [20–23, 25–29, 31, 32] have considered age in their analyses, thus the potential biasing influence of age could be minimized. Moreover, obesity during adolescence has been shown to be positively associated with prevalent hearing loss [42, 43].

The positive association between BMI and hearing loss could also be mediated or confounded by the cardiometabolic comorbidities related to excess weight. DM [14, 58, 59] and, to the lesser extent, CVD [15–17] and its cardiovascular risk factors [25, 26, 28] have all been shown to be positively associated with hearing loss. Unfortunately, we could not investigate whether the observed positive association between overweight/obesity is independent of DM, CVD, or cardiometabolic risk factors owing to the limited studies adjusted for these conditions. The Nurses’ Health Study II showed that BMI was associated with the risk of self-reported hearing loss in a dose-dependent manner even after DM and hypertension were taken into consideration [20], whereas the Health Professionals Follow-up found no such association after adjustment for DM, hypertension, and elevated cholesterol [28]. A cross-sectional data from the National Health and Nutrition Examination Survey, 2005 to 2010, showed that adolescent obesity was positively associated with prevalence of hearing loss independent of several cardiometabolic risk factors (high-density lipoprotein level, triglyceride level, elevated blood pressure measurement, hemoglobin A1c level, and C-reactive protein level) [41].

Adipose tissue may differently affect the health risk according to the site of deposition. It has been suggested that excessive accumulation of adipose tissue around the intra-abdominal organs, central adiposity, confers greater risk for cardiometabolic complications of obesity [61]. There is evidence that central adiposity may be more important than overall adiposity in predicting greater risk of cardiometabolic complications related to excess adiposity [62–64], although this is not always the case. As discussed, obesity and its cardiometabolic comorbidities may be relevant in the development of hearing loss. On the basis of the aforementioned rationales, WC, a proxy of central adiposity, is expected to be a better predictor for hearing loss than BMI, a proxy for overall adiposity. The findings from cross-sectional studies showed that both elevated BMI and higher WC were associated with poorer hearing thresholds [65–67]. Consistently, both elevated BMI and higher WC were positively associated with hearing loss. However, the relative contribution of BMI and WC to hearing loss has been rarely considered. Notably, only one study [21] on the association between BMI or WC and hearing loss has performed mutual adjustment of BMI and WC, and this study showed that high BMI (25 to > 40 kg/m^2^) and high WC (> 88 cm) were independently associated with an increased risk of hearing loss after mutual adjustment. Nonetheless, future studies need to investigate whether BMI and WC independently contribute to hearing loss.

It is also possible that excess weight and its cardiometabolic comorbidities may be the consequences of exposure to poor lifestyle choices that could also contribute to hearing loss. In other words, excess weight might be a proxy of unhealthy lifestyle habits. It is known that excess consumption of certain foods, particularly those that contain high amounts of refined grains, sugars, and trans fat, is associated with increased risks of obesity, DM, and CVD [68]. The Blue Mountains Hearing Study [69] showed that higher glycemic index was associated with an increased prevalence of hearing loss, whereas a higher dietary cereal fiber was inversely associated with the prevalence of hearing loss. Higher dietary glycemic load had a 76% higher odds of having hearing loss in all participants, while lower intake of carbohydrate and sugar was associated with a 62% and 72% lower odds of having hearing loss in the participants aged less than 70 years, respectively. Another interesting finding from the Blue Mountains Hearing Study [70] indicated that higher intake of dietary cholesterol was positively associated with prevalence of hearing loss, whereas treatment with statins and higher intake of dietary monounsaturated fats were inversely associated with prevalence of hearing loss. Of interest, a recent longitudinal cohort study of female nurses [71] suggests that adherence to the healthful dietary patterns that are often linked to a better cardiometabolic health was associated with a lower risk of hearing loss. Moreover, there is evidence that the incident [20] and prevalent [20, 72] of hearing loss is inversely associated with physical activity, a non-nutritional determinant for weight control that has been linked to a better cardiometabolic profile [73].

## Limitations

The present meta-analysis should be interpreted within the context of its limitations. First, the present meta-analysis pooled the data from the observational studies. Therefore, the unmeasured or residual confounders may also influence of the associations between BMI, WC, and the risk of hearing loss. For example, only few studies performed adjustment for important risk factors for hearing loss, such as longstanding noise exposure, medical conditions that may lead to hearing loss, and the use of ototoxic drugs. Moreover, we could not rule out the potential role of cardiometabolic comorbidities related to excess weight as mediators of the associations between BMI or WC and hearing loss. Second, we did not have enough data sets to investigate the associations between BMI or WC and hearing loss according to several important factors, such as type of hearing loss (sensorineural or age-related), frequency of hearing loss, and severity of hearing loss. The potential effect modification by these factors warrants further investigation. Third, slight variation in the cut-points (frequencies and threshold) used to define hearing loss among the included studies should also be considered. Finally, high heterogeneity observed across studies suggests that the findings should be treated with caution. Unfortunately, we were unable to pin-point the source of heterogeneity because subgroup analyses could not be performed. However, high heterogeneity appeared to be mainly caused by differences in the strength (significant vs. non-significant) of association across studies rather than differences in the direction (inverse vs positive) of association across studies as the vast majority of studies on elevated BMI (overweight and obesity) or higher WC showed a tendency toward positive associations, although some did not reach statistical significance.

## Conclusions

In summary, our findings add weight to the evidence that elevated BMI and higher WC may be positively associated with the risk of hearing loss. However, the nature of the observed positive associations remains unclear because we were unable to consider the type and severity of hearing loss, noise exposure, and other potential effect modifiers or mediators in our analyses. These issues need to be addressed by well-performed prospective studies.

## Supplementary information

**Additional file 1: Table S1.** Characteristics of the included studies.

**Additional file 2: Table S2.** The Newcastle Ottawa scale for cohort study.

**Additional file 3: Table S3.** The Newcastle Ottawa scale for cross-sectional study.

## Data Availability

The datasets used and analyzed in the present study are available from the corresponding author upon reasonable request.

## Abbreviations

BMI: Body mass index
CIs: Confidence intervals
DM: Diabetes mellitus
HRs: Hazard ratios
NOS: Newcastle-Ottawa Scale
ORs: Odds ratios
RRs: Relative risks
WC: Waist circumference
WHO: World Health Organization
