# Supplementary material for: Body mass index, waist circumference, and risk of hearing loss: a meta-analysis and systematic review of observational study
Source: Environ Health Prev Med. 2020 Jun 26;25:25. doi: 10.1186/s12199-020-00862-9 (PMC7320546; doi:10.1186/s12199-020-00862-9)
Supplement: Supplementary file 1 — Additional file 1: Table S1. Characteristics of the included studies. [file 12199_2020_862_MOESM1_ESM.docx]

**Table S1. Characteristics of the included studies.**

| Reference | Country | | Design | Age (mean) | Men, % | Total sample (no. cases) | Exposure category | Ascertainment of exposure | Hearing test method | Definition of hearing loss | | Adjustment |
| --- | --- | --- | --- | --- | --- | --- | --- | --- | --- | --- | --- | --- |
|  |  |  |  |  |  |  |  |  |  | Frequency | Threshold |  |
| Barrenas et al., 2005 (19) | Sweden | | Longitudinal, population-based | 17-24 | 100 | 245,092 (8,869) | BMI: <18.5 kg/m^2^, 18.5-24.9 kg/m^2^, 25-29.9 kg/m^2^, ≥30 kg/m^2^ | Measured | PTA | 1-6 kHz | >20 dB, average, in either ears | Birth length for GA, birth weight for GA, head circumference for GA, GA, and adult height |
| Shargorodsky et al., 2010 (29) | USA | | Longitudinal, population-based | 40-74 (51) | 100 | 26,917 (3,488) | BMI: <19 kg/m^2^, 19-24 kg/m^2^, 25-29 kg/m^2^, ≥30 kg/m^2^ | Self-reported | Self-reported professionally diagnosed hearing loss | NA | NA | Age, race, profession, smoking, hypertension, diabetes, elevated cholesterol, aspirin, NSAID, and acetaminophen use |
| Curhan et al., 2013 (20) | USA | | Longitudinal, population-based | 25-42 (34.5) | 0 | 68,421 (11,286) | BMI: <25 kg/m^2^, 25-29 kg/m^2^, 30-34 kg/m^2^, 35-39 kg/m^2^, ≥40 kg/m^2^  WC: <71 cm, 71-79 cm, 80-88 cm, >88 cm | Self-reported | Self-reported | NA | NA | Age, race, smoking, alcohol intake, intake of B12, magnesium, potassium, vitamin A, and folate, baseline hypertension, baseline diabetes, acetaminophen use, ibuprofen use, physical activity, BMI/WC |
| Sogebi et al., 2014 (30) | Nigeria | | Cross-sectional, hospital based | 45-94 (69.6) | 59.8 | 127 (76) | BMI: ≥30 kg/m^2^ | Measured | PTA | 0.5-8 kHz | >25dB, average, in both ears | None |
| Cruickshanks et al., 2015 (21) | USA | | Longitudinal, population-based | 43-84 (61) | 32.5 | 1,678 (835) | BMI: ≥30 kg/m^2^;  WC: per 10 cm | Measured | PTA | 0.5-8 kHz | >25dB, average, either ears | Age and sex; model for WC was additionally adjusted for educational status, current smoking, and high glycosylated hemoglobin (>12%) |
| Lohi et al., 2015 (22) | Finland | | Cross-sectional, population-based | 54-66 (60.9) | 100 | 352 (206) | BMI: ≥30 kg/m^2^ | Self-reported | PTA | 0.5-4 kHz | ≥20 dB, average, in both ear | Age, socioeconomic class, and smoking |
| Sumit et al., 2015 (31) | Bangladesh | | Cross-sectional, population-based | 18-60 (37.7) | 100 | 184 (104) | BMI: <18.5 kg/m^2^, 18.5-25 kg/m^2^, >25 kg/m^2^ | Self-reported | PTA | 1-4 kHz | ≥20 dB, average | Age and smoking |
| Jung et al., 2016 (32) | South Korea | | Cross-sectional, population-based | ≥65 (72) | 44 | 1,032 (542) | BMI for men: <21.9 kg/m^2^, 21.9-24.5 kg/m^2^, >24.6 kg/m^2^; BMI for women: <22.8 kg/m^2^, 22.8-25.6 kg/m^2^, >25.6 kg/m^2^ | Measured | PTA | 0.5-4 kHz | >25 dB, average, in the better ear | Age, mean daily alcohol intake, smoking, diabetes mellitus, hypertension, exposure to explosive noise, and exposure to occupational noise |
| Reference | | Country | Design | Age (mean) | Men, % | Total sample (no. cases) | Exposure category | Ascertainment of exposure | Hearing test method | Definition of hearing loss | | Adjustment |
|  |  |  |  |  |  |  |  |  |  | Frequency | Threshold |  |
| Kim et al., 2016 (23) | | South Korea | Cross-sectional, hospital based | (46.1) | 79.8 | 61,052 (12,538) | BMI: <18.5 kg/m^2^, ≥18.5 to <22.9 kg/m^2^, ≥23 to <24.9 kg/m^2^, ≥25 to <29.9 kg/m^2^, ≥30 kg/m2 | Measured | PTA | 0.5-4 kHz | >26 dB, average, in the both ears | Age, sex, and BMI status. |
| Aghazadeh-Attari et al., 2017 (24) | | Iran | Cross-sectional, population-based | 20-60 (39.7) | 100 | 11,114 (2,772) | BMI: <25 kg/m^2^, ≥25 kg/m^2^,  ≥30 kg/m^2^ | Measured | PTA | 4 kHz | >25 dB, in the better ear | Smoking |
| Tan et al., 2017 (25) | | Australia | Cross-sectional, population-based | 45-69 (58) | 45.5 | 4,877 (209) | BMI: <25 kg/m^2^, 25-29 kg/m^2^, 30-34 kg/m^2^, ≥35 kg/m^2^  WC: >94 cm in men；>80 cm in women | Measured | Self-reported | NA | NA | Age, sex, and family history of HL |
| Han et al., 2018 (26) | | China | Cross-sectional, population-based | (64.7) | 44.4 | 18,824 (9,839) | BMI: <18.5, 18.5-24, 24-28, ≥28  WC: ≥ 90 cm in men and ≥ 80 cm in women | Measured | PTA | 0.5-4 kHz | >25 dB, average, in the better ear | Model for WC was adjusted for age, sex, smoking status and alcohol consumption status plus family history of deafness, use of ototoxic drugs (gentamicin, streptomycin, and kanamycin), history of occupational noise exposure, and components of the metabolic syndrome; Model for BMI was adjusted for the same variables in the model for WC plus central obesity |
| Wang et al., 2018 (27) | | Australia | Longitudinal, population-based | (43.4) | 0 | 1,135 (not specified) | BMI: <18.5 kg/m^2^, 18.5-24.9 kg/m^2^, 25-29.9 kg/m^2^, 30-34.9 kg/m^2^, ≥35 kg/m^2^ | Measured | PTA | 1-4 kHz | >25 dB, average, in the better ear | Age and neighborhood disadvantage |
| Hu et al., 2019 (28) | | Japan | Longitudinal, population-based | 20-64 (39.1) | 84.9 | 48,549 (4,646) | BMI: <25 kg/m^2^, 25-29 kg/m^2^, ≥30 kg/m^2^  WC: ≥ 90 cm in men and ≥ 80 cm in women | Measured | PTA | 1, 4 kHz | >30 dB at 1kHz or >40 dB at 4kHz | Age, sex, worksite, smoking, cardiovascular disease, diabetes, hypertension, dyslipidemia alcohol intake, leisure time physical activity and occupational noise exposure |

BMI, body mass index; GA, gestational age; NA, not applicable; NSAIDs, Nonsteroidal anti-inflammatory drugs; PTA pure tone auditory; WC, waist circumference
