# Supplementary material for: Body mass index, waist circumference, and risk of hearing loss: a meta-analysis and systematic review of observational study
Source: Environ Health Prev Med. 2020 Jun 26;25:25. doi: 10.1186/s12199-020-00862-9 (PMC7320546; doi:10.1186/s12199-020-00862-9)
Supplement: Supplementary file 2 — Additional file 2: Table S2. The Newcastle Ottawa scale for cohort study. [file 12199_2020_862_MOESM2_ESM.docx]

**Table S2.** The Newcastle Ottawa scale for cohort study

| Study | **Selection** | | | | **Comparability** | **Outcome** | | | **Total stars** |
| --- | --- | --- | --- | --- | --- | --- | --- | --- | --- |
|  | Representativeness of the exposed cohort | Selection of the non-  exposed  cohort | Ascertainment of exposure | Demonstration that outcome of interest was not present at start of study | Comparability  of cohorts on the basis of the design or analysis** | Assessment of outcome | Was follow-  up long enough for  outcomes to  occur | Adequacy  of follow  up of  cohorts |  |
| Barrenas et al., 2005 (19) | 1 | 1 | 1 | 1 | 0 | 1 | 1 | 1 | 7 |
| Shargorodsky et al., 2010 (29) | 1 | 1 | 0 | 1 | 2 | 0 | 1 | 1 | 7 |
| Curhan et al., 2013 (20) | 1 | 1 | 0 | 1 | 2 | 0 | 1 | 1 | 7 |
| Cruickshanks et al., 2015 (21) | 1 | 1 | 1 | 1 | 2 | 1 | 1 | 1 | 9 |
| Wang et al., 2018 (27) | 1 | 1 | 1 | 1 | 1 | 1 | 1 | 1 | 8 |
| Hu et al., 2019 (28) | 1 | 1 | 1 | 1 | 2 | 1 | 1 | 1 | 9 |

**A maximum of two stars can be awarded.
