# Supplementary material for: Body mass index, waist circumference, and risk of hearing loss: a meta-analysis and systematic review of observational study
Source: Environ Health Prev Med. 2020 Jun 26;25:25. doi: 10.1186/s12199-020-00862-9 (PMC7320546; doi:10.1186/s12199-020-00862-9)
Supplement: Supplementary file 3 — Additional file 3: Table S3. The Newcastle Ottawa scale for cross-sectional study. [file 12199_2020_862_MOESM3_ESM.docx]

**Table S3.** The Newcastle Ottawa scale for cross-sectional study

| Study | **Selection** | | | | **Comparability** | **Outcome** | | **Total stars** |
| --- | --- | --- | --- | --- | --- | --- | --- | --- |
|  | Representativeness of the sample | Sample size | Non-respondents | Ascertainment of the exposure** | Comparability of subjects in different outcome groups on the basis of design or analysis** | Assessment of outcome** | Statistical test |  |
| Sogebi et al., 2014 (30) | 1 | 1 | 0 | 2 | 0 | 2 | 1 | 7 |
| Lohi et al., 2015 (22) | 1 | 1 | 1 | 0 | 2 | 2 | 1 | 8 |
| Sumit et al., 2015 (31) | 1 | 1 | 0 | 0 | 2 | 2 | 1 | 7 |
| Jung et al., 2016 (32) | 1 | 1 | 0 | 2 | 2 | 2 | 1 | 9 |
| Kim et al., 2016 (23) | 1 | 1 | 0 | 2 | 2 | 2 | 1 | 9 |
| Aghazadeh-Attari et al., 2017 (24) | 1 | 1 | 0 | 2 | 0 | 2 | 1 | 7 |
| Tan et al., 2017 (25) | 1 | 1 | 0 | 2 | 1 | 0 | 1 | 6 |
| Han et al., 2018 (26) | 1 | 1 | 0 | 2 | 2 | 2 | 1 | 9 |

**A maximum two stars can be awarded.
